# Supplementary figures and images for: Preventable risk factors for type 2 diabetes can be detected using noninvasive spontaneous electroretinogram signals
Source: PLoS One. 2023 Jan 12;18(1):e0278388. doi: 10.1371/journal.pone.0278388 (PMC9836271; doi:10.1371/journal.pone.0278388)

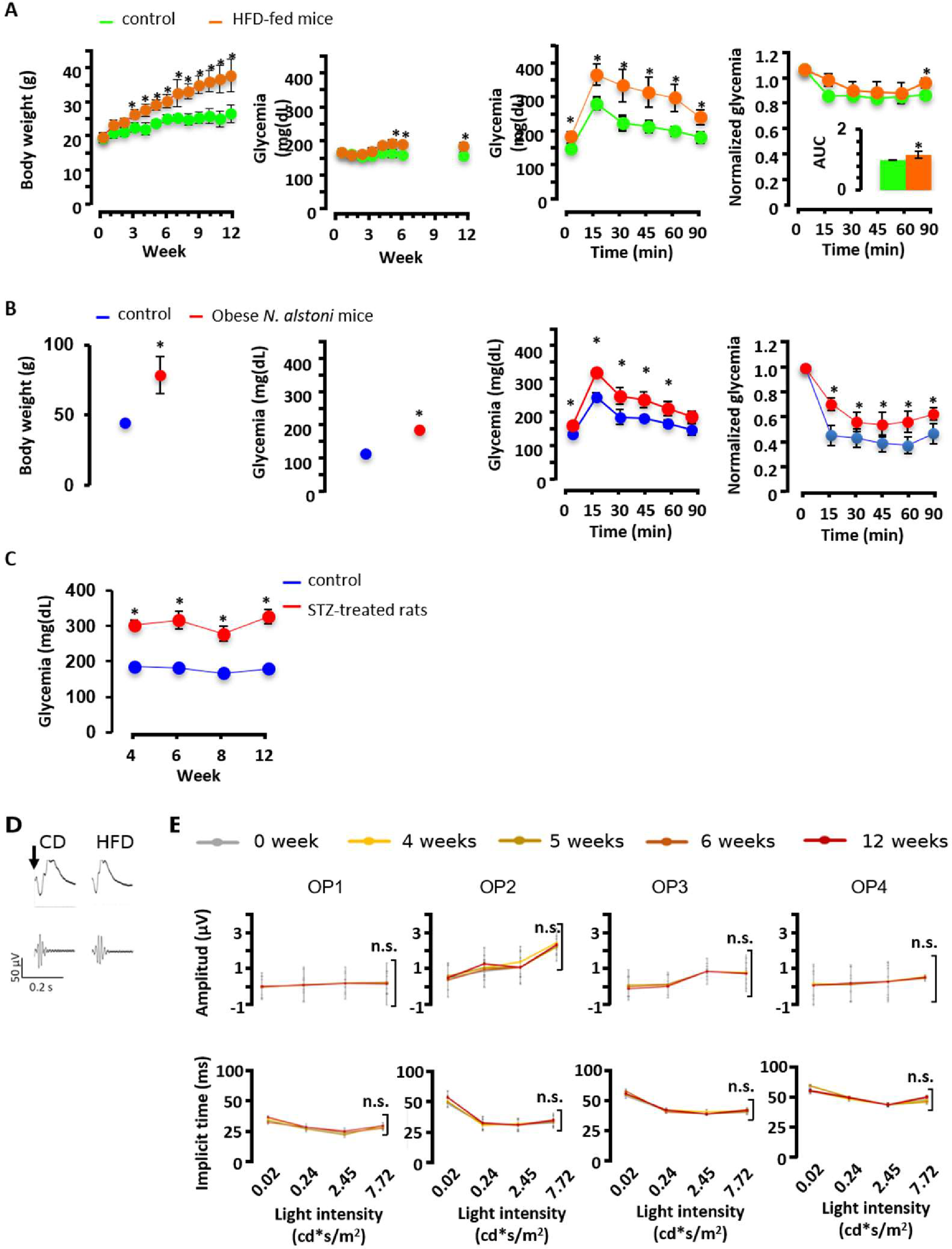

Supplement: S1 Fig — A, Follow-up of body weight and blood glucose levels, glucose tolerance test, and insulin tolerance test in control-diet (n = 75) and high-fat diet-fed (n = 75) mice for 12 weeks. B, Body weight, blood glucose levels, glucose tolerance test, and insulin tolerance test in lean (n = 20) and spontaneously obese (n = 20) Neotomodon alstoni mice. In both models, mice have higher glycemia than control mice at every time point (P < 0.05), suggesting reduced insulin sensitivity. This was confirmed by insulin tolerance tests, which showed a lower fall in blood glucose in response to insulin in obese mice as compared with control mice. C, Blood glucose level follow-up in rats after 4, 6, 8, or 12 weeks of streptozotocin (n = 40) and vehicle (n = 40) treatment. Values, mean ± s.d. * indicates P values < 0.05 determined by a two-sample Student’s t-test in B (body weight and glycemia) and by a mixed ANOVA followed by Bonferroni test everywhere else. D, Illustrative ERG (top) and oscillatory potentials (OP, bottom) in control-diet and high-fat diet-fed mice for 12 weeks, measured in response to a light flash of 7.72 (cd.s)/m2 (arrow) under dark-adapted conditions. E, Temporal monitoring (0 to 12 weeks, as indicated) of the average amplitude and implicit time of OP1, OP2, OP3, and OP4 in control (n = 10) and high-fat diet fed mice (n = 12) under dark-adapted conditions at increasing light intensities (0.02, 0.24, 2.45, and 7.72 (cd.s)/m2. CD, control diet. HFD, high-fat diet. n.s., not significant (P > 0.05). (TIF) [file pone.0278388.s001.tif]

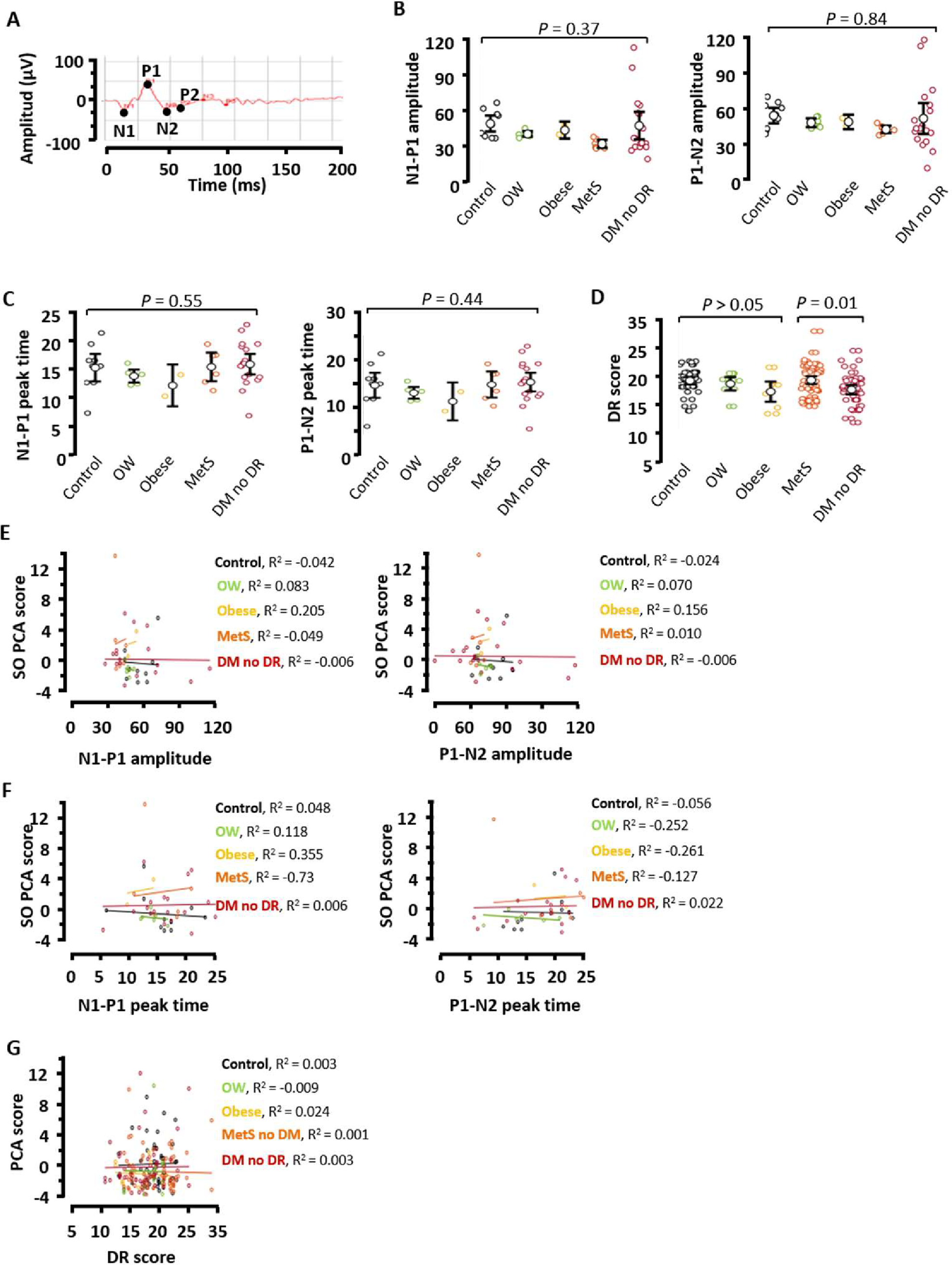

Supplement: S2 Fig — A, Illustrative recording from the ISCEV DA 3 ERG protocol in a control case. N1-P1 and P1-N2 B, amplitude and C, peak time ratio in control (metabolically healthy, n = 8) and disease (OW, n = 5; obese, n = 2; MetS, n = 6; and diabetes with no DR, n = 61) groups. D, DR score in control (metabolically healthy, n = 74) and disease (OW, n = 22; obese, n = 18; MetS, n = 88; and diabetes with no DR, n = 109) groups. B-D, Graphs show mean ± confidence interval. Correlation analysis between the E, N1-P1 or P1-N2 amplitude ratio, F, N1-P1 or P1-N2 peak time ratio, and G, DR score with the spontaneous oscillation (SO) PCA score (detailed in Methods) in the groups of interest. (TIF) [file pone.0278388.s002.tif]

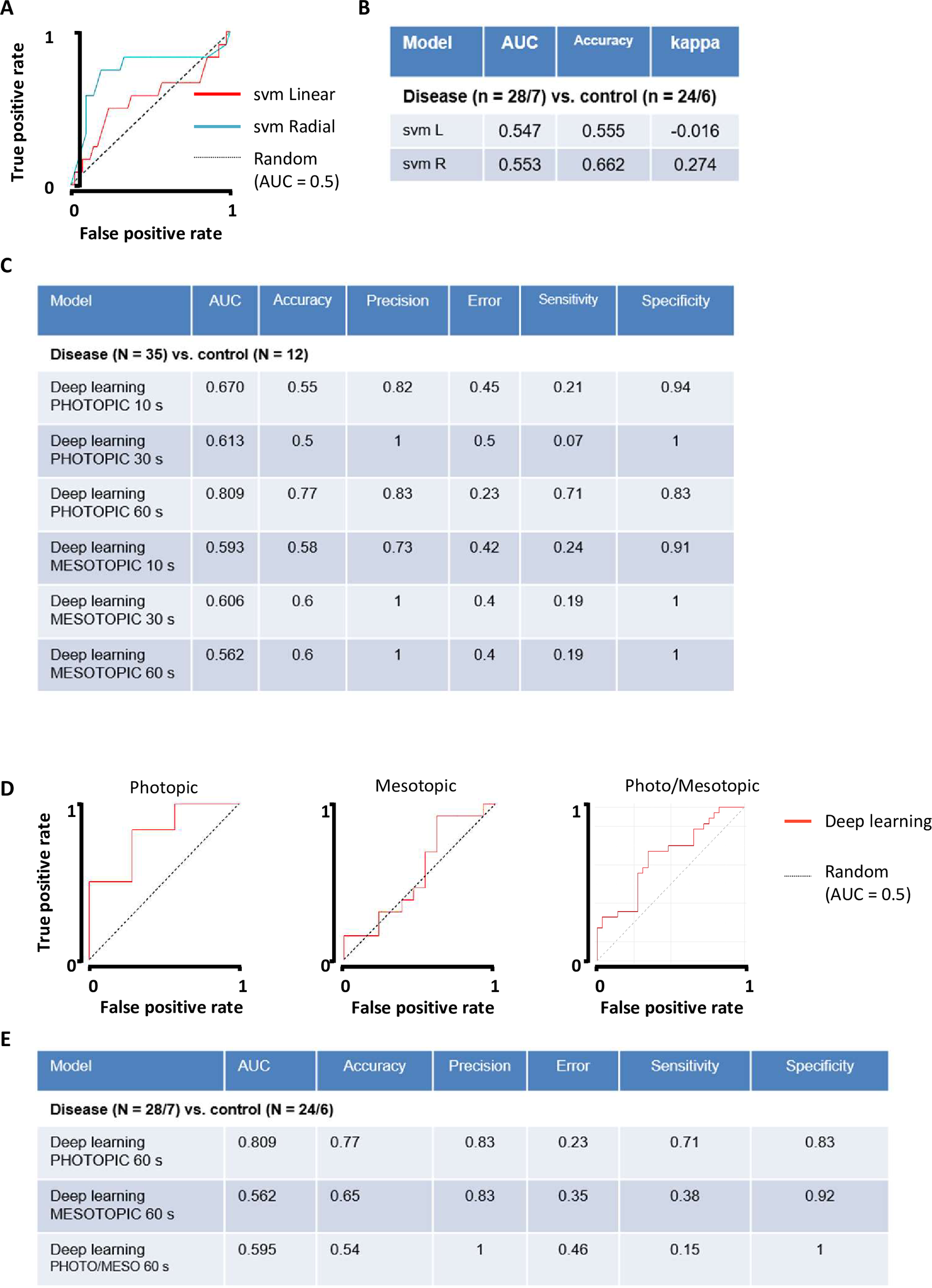

Supplement: S3 Fig — A, ROC curves for both linear and radial svm algorithms. B, Performance parameters for the random forest model using power spectra from photopic or mesopic ERGs of 10, 30 or 60 s. C, ROC curves for the random forest model using power spectra from photopic, mesopic or combined photopic and mesopic ERGs of 60 s. D, Corresponding performance parameters. All data correspond to binary classification between control and disease cases. Controls are constituted by metabolically healthy subjects (n = 62) and the disease group by patients with overweight (n = 41), obesity (n = 16), metabolic syndrome (n = 55), and diabetes with no DR (n = 63). (TIF) [file pone.0278388.s003.tif]
