# Supplementary material for: Preventable risk factors for type 2 diabetes can be detected using noninvasive spontaneous electroretinogram signals
Source: PLoS One. 2023 Jan 12;18(1):e0278388. doi: 10.1371/journal.pone.0278388 (PMC9836271; doi:10.1371/journal.pone.0278388)
Supplement: Attachment — Submitted filename: Response to reviewer.docx [file pone.0278388.s004.docx]

Response to editor/reviewers

PONE-D-22-22890R1

Title: Preventable risk factors for type 2 diabetes can be detected using noninvasive spontaneous electroretinogram signals

Authors’ list: Ramsés Noguez Imm^*^, Julio Muñoz-Benitez^*^, Diego Medina, Everardo Barcenas, Guillermo Molero-Castillo, Pamela Reyes-Ortega, Jorge Armando Hughes-Cano, Leticia Medrano-Gracia, Manuel Miranda-Anaya, Gerardo Rojas-Piloni, Hugo Quiroz-Mercado, Luis Fernando Hernández-Zimbrón, Elisa Denisse Fajardo-Cruz, Ezequiel Ferreyra-Severo, Renata García-Franco, Juan Fernando Rubio Mijangos, Ellery López-Star, Marlon García-Roa, Van Charles Lansingh, and Stéphanie C. Thébault.

**ACADEMIC EDITOR: Please re-order the manuscript's structure into: Abstract - Introduction - Methods - Results - Discussions - Conclusion. Thank you!**

**Answer:** Dear Tri Juli Edi Tarigan, the Ms sections have been reordered as indicated.

**1. Please ensure that your manuscript meets PLOS ONE's style requirements, including those for file naming. The PLOS ONE style templates can be found at** 
[**https://journals.plos.org/plosone/s/file?id=wjVg/PLOSOne_formatting_sample_main_body.pdf**](https://journals.plos.org/plosone/s/file?id=wjVg/PLOSOne_formatting_sample_main_body.pdf) **and** 
[**https://journals.plos.org/plosone/s/file?id=ba62/PLOSOne_formatting_sample_title_authors_affiliations.pdf**](https://journals.plos.org/plosone/s/file?id=ba62/PLOSOne_formatting_sample_title_authors_affiliations.pdf)

**Answer:** Done

**2. To comply with PLOS ONE submissions requirements, in your Methods section, please provide additional information regarding the experiments involving animals and ensure you have included details on (1) methods of sacrifice, and (2) efforts to alleviate suffering.**

**Answer:** The method of sacrifice has been indicated (p.6, line 115) as well as efforts to alleviate animal suffering (p.5, lines 100-101; p.7, lines 131-132 and lines 135-137).

**3. Please include your tables as part of your main manuscript and remove the individual files. Please note that supplementary tables (should remain/ be uploaded) as separate "supporting information" files.**

**Answer:** Done and acknowledged.

**4. We note that the grant information you provided in the ‘Funding Information’ and ‘Financial Disclosure’ sections do not match.**

**Answer:** Thank you, we amended the ‘Funding information’ section.

**5. We noted in your submission details that a portion of your manuscript may have been presented or published elsewhere. [**[**https://doi.org/10.1101/2022.06.26.22276881**](https://doi.org/10.1101/2022.06.26.22276881)**] Please clarify whether this [conference proceeding or publication] was peer-reviewed and formally published. If this work was previously peer-reviewed and published, in the cover letter please provide the reason that this work does not constitute dual publication and should be included in the current manuscript.**

**Answer:** Our manuscript was indeed posted as a pre-print in MedRxiv **[**[**https://doi.org/10.1101/2022.06.26.22276881**](https://doi.org/10.1101/2022.06.26.22276881)**]**. As preprints are preliminary reports of work that have not been certified by peer review, we consider that this work does not constitute a case of dual publication.

**6. Please review your reference list to ensure that it is complete and correct. If you have cited papers that have been retracted, please include the rationale for doing so in the manuscript text, or remove these references and replace them with relevant current references. Any changes to the reference list should be mentioned in the rebuttal letter that accompanies your revised manuscript. If you need to cite a retracted article, indicate the article’s retracted status in the References list and also include a citation and full reference for the retraction notice.**

**Answer:** Our reference list is correct and up to our knowledge, none of the cited papers have been retracted.
